# The effect of tissue surface modification with collagenase and addition of TGF-β3 on the healing potential of meniscal tears repaired with tissue glues in vitro

**DOI:** 10.1007/s10856-016-5832-0

**Published:** 2016-12-26

**Authors:** Agnieszka Izabela Bochyńska, Gerjon Hannink, Renate Verhoeven, Dirk W. Grijpma, Pieter Buma

**Affiliations:** 1 Orthopaedic Research Laboratory, Department of Orthopaedics, Nijmegen Center for Molecular Life Sciences, Radboud University Medical Center, Nijmegen, The Netherlands; 2 MIRA Institute for Biomedical Engineering and Technical Medicine, Department of Biomaterials Science and Technology, University of Twente, Enschede, The Netherlands; 3 W.J. Kolff Institute, Department of Biomedical Engineering, University Medical Centre Groningen, University of Groningen, Groningen, The Netherlands

## Abstract

The aim of the current in vitro study was to investigate if tissue surface modification with collagenase and addition of the TGF-β3 can increase the number of cells present in meniscus tears repaired with the use of newly developed tissue adhesives based on isocyanate-terminated block copolymers. Cylindrical explants were harvested from the inner part of bovine menisci. To simulate a full-thickness tear, the central core of the explants was removed and glued back into the defect, with or without incubation in collagenase solution prior to gluing. The repair constructs were then cultured with or without addition of TGF-β3, and assessed for their histological appearance. The histological staining of the constructs confirmed that both developed adhesives were not cytotoxic. After 28 days, meniscus cells were present in direct contact with the glues. The addition of TGF-β3 to the culture medium resulted in the presence of cells that formed a sheath inside the simulated tear and in increased cell numbers at the edges of annulus of the explants. In the group in which the tissue was incubated in collagenase and cultured in medium containing TGF-β3, thicker layers of cells were observed. These results suggest that repairing the torn meniscus with tissue adhesives after pre-treatment of the tissue with collagenase and stimulation with TGF-β3 is a very promising treatment method, especially when treating the inner avascular part of the meniscus. Nevertheless, longer-term in vitro and in vivo studies are needed to confirm the beneficial effects of this combination therapy.

## Introduction

Biodegradable tissue adhesives are an attractive class of materials with potential to be used in the repair of meniscus tears [1–3]. They are relatively easier to apply than sutures, and would be less expensive. The glue would keep the torn parts of the tissue together during the period of healing, and degrade into harmless compounds that are then metabolized or excreted from the body.

However, meniscus tissue is largely avascular and its dense extracellular matrix (ECM) restricts the already relatively low number of cells in their mobility and proliferation potential [4, 5]. Hence, the capacity of the meniscus for self-repair is very limited and tears, especially those located in the avascular zone, do not heal spontaneously. In clinical practice, rasping of the tissue and trephination to create access channels to stimulate neo-vascularization and new tissue ingrowth is an often-performed procedure [6–9]. Still, the success rate of repairing tears in the avascular zone is disappointing. The study of Rubman et al. showed that out of the 91 meniscal repairs evaluated arthroscopically, 23 (25%) were classified as healed, 35 (38%) as partially healed, and 33 (36%) as failed [10]. 20% of treated patients reported on in this study required a second intervention [10]. Therefore, there is a rapidly growing interest in the use of biological factors that could induce meniscus repair [11]. Qu et al. showed that partial digestion of adult meniscal explants with collagenase prior to culture in vitro resulted in a less dense ECM at the edges of the explants and in an increased cellularity [12]. A later in vivo experiment using ovine menisci confirmed that partial digestion of the tissue enhances repair by creating a more compliant and porous microenvironment that facilitates migration of cells to the tear margin and their proliferation [13]. Another approach to stimulate cellular migration and proliferation is the local delivery of growth factors [14–16]. Particularly, transforming growth factor TGF-β3, which is an anabolic factor for meniscus fibroblasts, was shown to increase the integration strength in both adult and juvenile menisci in vitro [15].

We recently reported on the development of reactive three-armed and hyper-branched block copolymeric tissue adhesives that can attach to the tissue via covalent bonding [17, 18] In a following study, meniscus explants were glued with these adhesives and cultured in vitro. It was shown that both developed materials have satisfactory bonding strengths to the tissue and did not exhibit toxicity to the cells, and therefore have the potential to be used in the treatment meniscal tears (unpublished work). However, the continuous glue layer present in the repaired meniscal tear might limit cell migration and the transport of nutrients and waste products.

The aim of the current study was twofold. First, to evaluate if partial gluing of the repaired tissue allows cells to migrate into the simulated tear (gap). Second, to investigate if modification of the tissue surface with collagenase and the addition of TGF-β3 can increase the number of cells present in the repaired meniscus tear and thereby enhance healing of the tissue. We hypothesized that the optimal treatment for meniscus tissue repair would be a degradable glue to hold the edges of meniscal tear in close proximity to each other, in combination with additional biological factors to enhance the capacity of the meniscus for self-repair.

## Materials and methods

### Tissue glues

Two different reactive isocyanate-terminated adhesive block copolymers were prepared: a three-armed TMPE-(TMC_2_-HDI)_3_ adhesive and a hyper-branched CA-4PEG-(TMC_2_)_2_-HDI adhesive. Their synthesis has been described in detail before [17, 18]. These adhesives were respectively prepared from trimethylolpropane ethoxylate (TMPE, *M*_n_ = 450 g/mol), trimethylene carbonate (TMC) and hexamethylene diisocyanate (HDI), and from polyethylene glycol (PEG, *M*_n_ = 400 g/mol), TMC, citric acid (CA) and HDI. Prior to use, the adhesives were sterilized by gamma irradiation (25 kGy, SynergyHealth BV, The Netherlands). The materials were characterized by ^1^H-NMR (Bruker 400 MHz NMR spectrometer) and Fourier transform infrared spectroscopy (FTIR) (Perkin Elmer) before and after sterilization.

### Preparation of meniscus explants

Meniscus explants were prepared in an analogous way as described by Hennerbichler et al. [19, 20]. Briefly, fresh knees from 12–18 months old cows were purchased from a local slaughterhouse. The medial menisci were removed from the joint under sterile conditions. Five cylindrical explants (8 mm-diameters) were obtained from the central part of each meniscus with a biopsy punch and randomly assigned to different experimental groups. Subsequently, their height was trimmed to 4 mm and an inner cylinder of 4 mm in diameter was centrically cored from each explant.

### The influence of surface modification with collagenase and TGF-β3 addition on cellular migration in glued meniscus tissue constructs

**Fig. 1:**
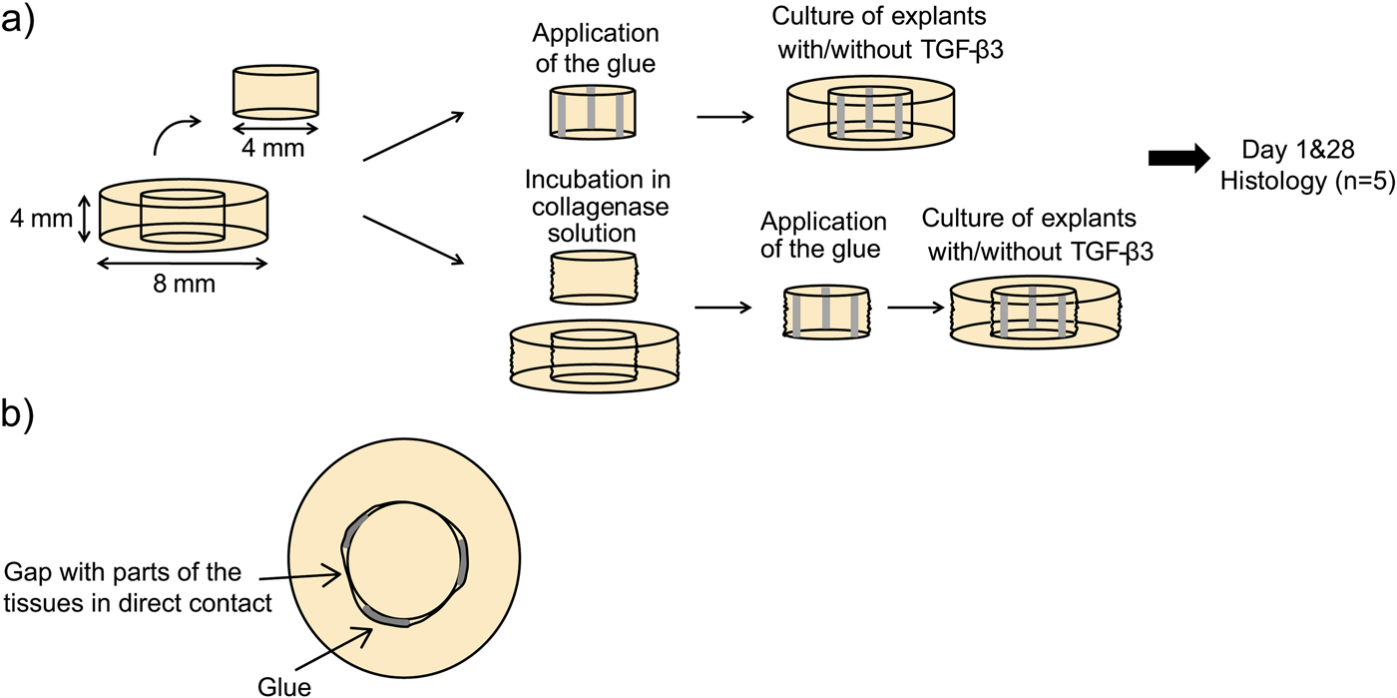
**a** An overview of the experimental design, **b** Top view on the meniscal repair construct glued with the strips of the glue

Explants (both outer annulus and inner cylinder) were incubated in basal medium containing 0 (control) or 0.05 mg/mL collagenase (type IV from *Clostridium histolyticum*, ≥125 collagenase digestion units/mg solid, Sigma Aldrich) for 3 h at 37 °C according to the protocol described by Qu et al. [13]. Basal medium was composed of Dulbecco’s Modified Eagle’s Medium (DMEM, Gibco®, UK) with 10% fetal bovine serum (FBS, Gibco®, UK), 1% penicillin/streptomycin/fungizone (PSF, Gibco®, USA) and L-ascorbic acid (Merck, Darmstadt, Germany) at a concentration of 25 µg/mL of medium. Repair constructs were created by applying three lines of glue (approximately 5–10 μL per line) onto the sides of the inner cylinder using a syringe equipped with a 23G needle and gently reinserting the inner cylinder into the outer annulus (Fig. 1a). This gluing procedure allowed parts of the tissue from inner cylinder and the outer annulus to remain in direct contact (Fig. 1b). Repair constructs were created using the three-armed adhesive and the hyper-branched one; constructs without any glue were used as controls.

Constructs were cultured for 1 and 28 days in basal medium containing 0 (control) or 10 ng/mL of TGF-β3. In total, four different culture conditions were employed (Table 1). After 1 and 28 days, the repair constructs (*n* = 5 per group) were fixated in 10% formalin, embedded in poly(methyl methacrylate) (PMMA), sectioned into 8 µm thick coupes and stained with hematoxylin and eosin (H&E) and picrosirius red (PR) to visualize cells, ECM and collagen fibers. For each group, H&E-stained sections were scored for the number of cells present inside and at the edge of the simulated meniscal tear, which was defined as a gap between inner cylinder and outer annulus of the explants. Moreover, the number of cells and the thickness of the cell layers at the outer edge of the annulus of the explants (no cells, single cell layer or multiple cell layers) were assessed. Digestion of collagen fibers was visually assessed on the PR stained sections. For the scoring, 5 explants per group at 3 random locations of each explant were analyzed by light microscopy (200X field magnification).

**Table 1:** Conditions used in the in vitro culturing of glued meniscus tissue constructs

Conditions	I	II	III	IV
Incubation in collagenase prior to gluing	no	yes	no	yes
Addition of TGF-β3 to the culture medium	no	no	yes	yes

### Statistical analysis

Differences in the number of cells in the different experimental groups were evaluated using a three-way ANOVA followed by Fischer’s LSD post-hoc test. Data are presented as a mean value ± standard deviation. *P* values < 0.05 were considered as significant. All statistical analyses were done using SPSS (version 20, IBM Corporation).

## Results

The reactive three-armed TMPE-(TMC_2_-HDI)_3_ and hyper-branched CA-4PEG-(TMC_2_)_2_-HDI adhesives were successfully synthesized as confirmed by ^1^H-NMR and FTIR analysis. No differences were observed before and after the sterilization procedure by gamma irradiation.

**Fig. 2:**
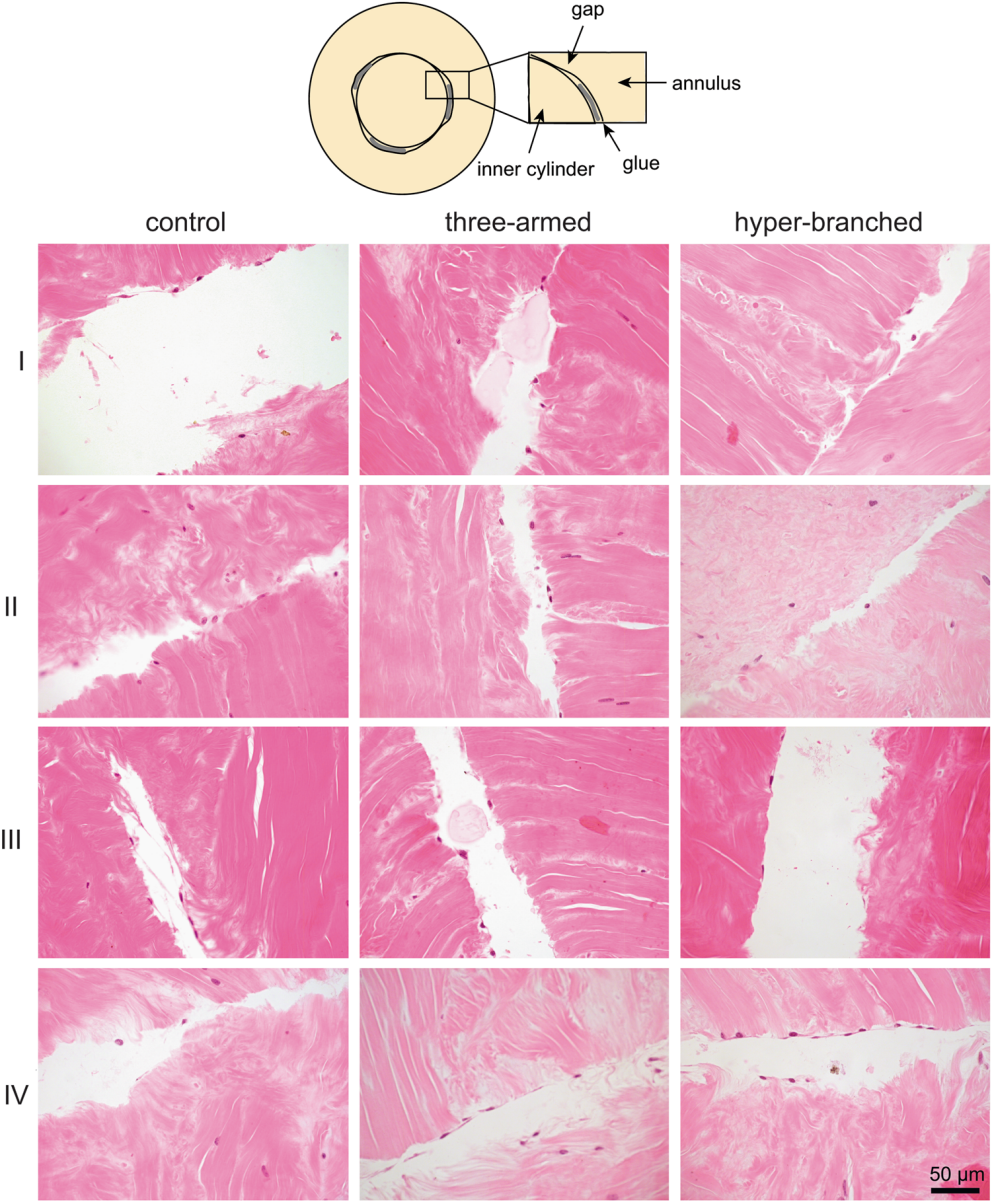
Representative light microscopy images of H&E stained meniscus tissue repair constructs cultured in vitro for 28 days under the different conditions (I-IV) described in Table 1. View on the partial gap between the inner cylinder and the annulus

The H&E staining showed that the glues did not induce any toxic reaction to the tissue, the tissue remained vital during the whole culturing period. After 1 day of explant culture, vital meniscus fibroblasts were seen in close proximity to the gap in the HE stained sections of all experimental groups. After 28 days cells could also be observed within the gap in all experimental groups. Within the same culturing conditions, there were no significant differences in cell numbers between the three-armed glue, the hyper-branched glue and the control group (Fig. 2). There was also no difference in the number of cells between conditions I and II, indicating that modification of the tissue surface by incubation in collagenase solution prior to culture did not have any beneficial influence on migration of the cells into the gap. However, there were differences between conditions IV, where explants were incubated in collagenase and TGF-β3 was added to the culture medium, and the other culturing conditions. Although the differences in the number of cells were not statistically significant between these different culturing conditions (after 28 days, respectively 3.6 ± 1.6, 3.7 ± 1.9 and 4.1 ± 1.7 for the three-armed glue, the hyper-branched glue and the control), it could clearly be seen that when culturing under conditions IV the cells populating the gap between the inner cylinder and the outer annulus start to form sheaths in the spaces between the lines of glue. This was never observed when culturing under the other conditions.

**Fig. 3:**
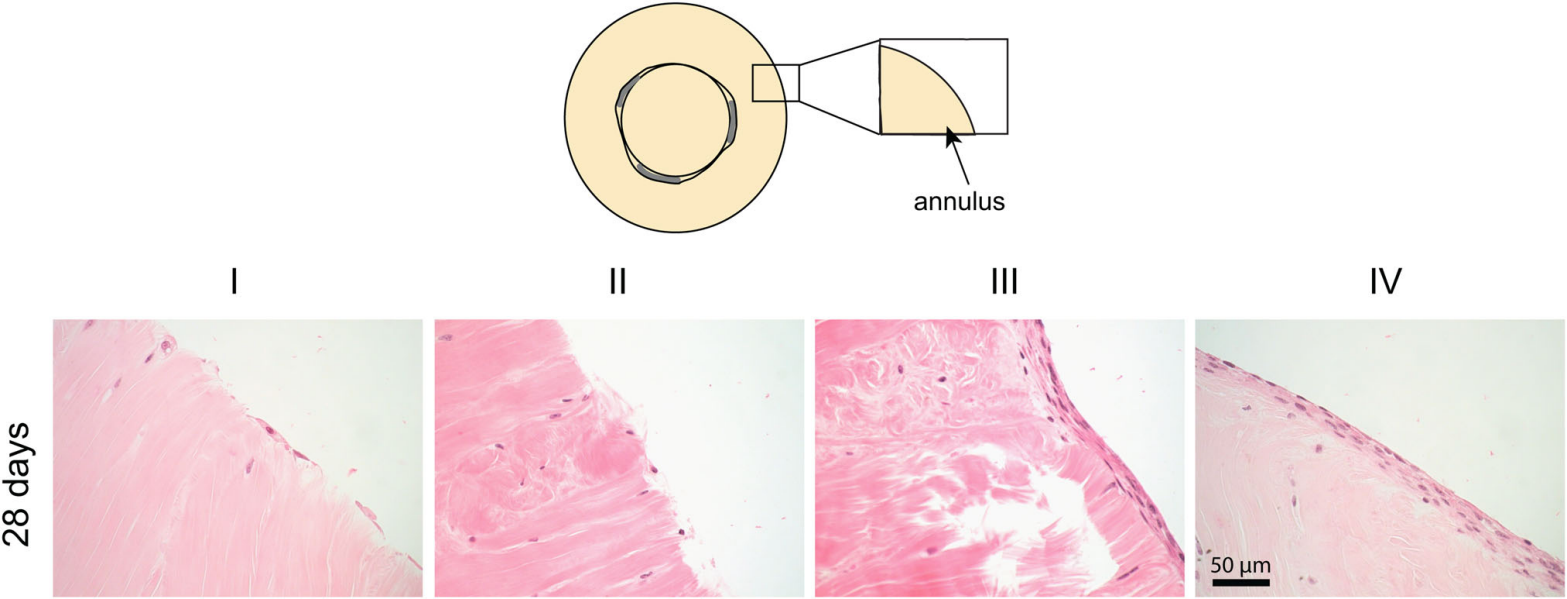
Typical images of the outer edge of the annulus of the repair constructs (H&E staining) cultured for 28 days under different conditions (I-IV) as described in Table 1. The figure shows images of the experimental three-armed glue group. In the other experimental groups (the hyper-branched glue and control groups), similar results were observed

At the outer edges of the annuli of the repair constructs there were no differences in cells numbers between the three-armed glue, the hyper-branched glue and the control group when cultured under the same conditions after 28 days (Fig. 3). However, the influence of incubation in collagenase solution and the addition of growth factor TGF-β3 was clearly visible (Fig. 3). When non-treated explants were cultured in medium containing TGF-β3 (III), single or double layers of cells aligned in an organized circumpherential manner could be seen. When explants were incubated in collagenase and cultured in medium supplemented with TGF-β3 (IV), even thicker multi-layered cell sheets were formed. This was not observed in explants cultured in the absence of growth factor (I and II), here only few cells at the outer edge of the repair constructs could be observed.

**Fig. 4:**
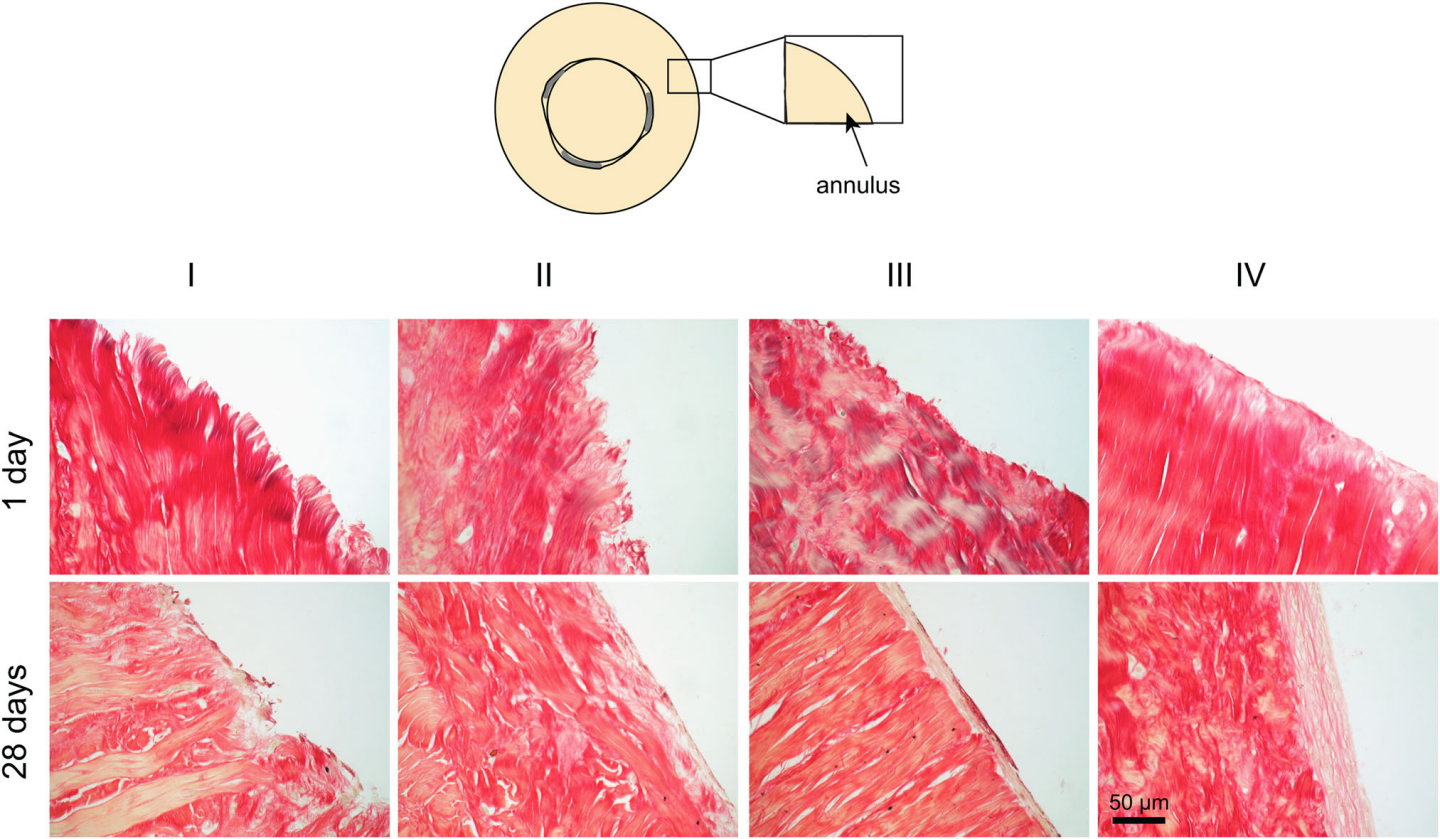
Typical images showing the alignment of collagen fibers (PR staining) at the outer edge of the annulus of the repair constructs after 1 and 28 days of culture under the different conditions (I-IV) described in Table 1. Images of repair constructs prepared using the three-armed glue are shown. In the other experimental groups (the hyper-branched glue and the control group), similar results were observed

Visualization of the collagen fibers at the outer edges of the repair constructs after 1 day of culturing (after PR staining) revealed that in the groups incubated in collagenase (II and IV) the constructs were partially digested (Fig. 4). After 28 days of culture, the influence of incubation in collagenase solution was even more pronounced. In the groups where TGF-β3 was added to the culture medium (III and IV) circumferentially oriented collagen fibers were detected. This was not observed in experimental groups I and II. In the experimental group where the explants were incubated in collagenase and the medium contained TGF-β3 (IV), the cellular layer and the collagen layer were thicker than in the group that only contained the growth factor (III).

## Discussion

The two recently developed tissue adhesives appeared to be promising materials for meniscus tears treatment. Clinical application of the glues on human menisci would be performed during arthroscopy in air. Using a small tip, a few small drops of the glue will be delivered into the tear and spread over the surfaces. In many cases, this procedure would be easier than the currently used gold standard, i.e., suturing. Growth factors or other biological active molecules could be incorporated in the glue. The procedure would require blotting of the surface of the meniscus tear with a tissue just prior to application of the glue to ensure good contact between the glue and the tissue. Once the glue is deposited on the tissue, it immediately starts curing without the need of any additional curing agents.

If the entire tear gap between the inner core and outer annulus of the explants is filled with glue, it could potentially limit cell migration. Therefore, in this study only partial gluing of the explants was performed. Such design will probably also be closer to a future clinical application, where only a few drops of glue would be applied to repair the tear [17]. In the present study, thin lines of the glues were applied to the tissue, and great care was taken to gently reinsert the inner cylinder into the outer annulus of the explants without spreading the glue over a larger surface area. As the glues could be seen in the histological slides after 1 and 28 days, partial gluing of the tissue, which stayed in place during the whole culture period, could be confirmed.

Histological evaluation of the gaps between the glued tissues in the repair constructs after 1 and 28 days showed the presence of fibroblasts in all experimental groups. Within 28 days of culture, cells were able to populate the gaps in between the lines of glue. There were no differences in cell numbers within the same culturing conditions, confirming the cell compatibility of the three-armed adhesive as well as that of the hyper-branched one.

The incubation of explants in collagenase and further addition of the TGF-β3 to the culture medium resulted in a presence of sheets of cells in the gap of the repair constructs. Indeed, TGF- β3 is known to be an anabolic chemotactic and mitogenic agent which attract cells and stimulates their replication [21]. Our findings are in accordance with data previously shown in literature that reprogramming the torn region of cartilaginous tissues such as the meniscus [12, 13, 15] and the articular cartilage [22, 23] with the use of enzymes, e.g., collagenase or hyaluronidase increases the density of cells at the wound edges and promotes repair.

Although sheets of cells were formed in the gap and the cells were found to deposit new ECM, complete bridging of the tear gap was never observed. The reason for this could be that the growth factor was immediately absorbed in the peripheral part of the tissue. As this part was in a direct contact with the medium, the influence of the growth factor on the inner part of the repair constructs might not have been as pronounced. After longer culturing times of the explants (e.g., 6 or 8 weeks), the cell layers in the gap could possibly be more extensive [15]. Moreover, even though only partial gluing of explants was performed, the two edges of the tear stayed in very close proximity to each other, probably limiting the amount of nutrient containing medium that is available for the cells present in the gap. Furthermore, meniscus tissue is a tissue with very limited numbers of cells, especially in the inner region from where the explants used in this study were obtained. This might also be a reason for the low cell numbers in the gap and the limited differences between the various culture conditions.

Investigation of the outer edge of annulus of the explants, where the cells likely had better access to TGF-β and nutrients in the culture medium, confirmed the positive influence of incubation in collagenase solution and the addition of TGF-β3 to the medium. Multi-layer cell sheets were present when the explants were both incubated in collagenase and cultured in medium containing growth factor. Slightly thinner layers were seen when only TGF-β3 was added to the medium. As the influence of both treatments was more pronounced at the outer edge of the annulus of the explants, it seems that the restricted access of medium to the gap is a limiting factor in our experimental setup. In clinical application, however, the glue will be applied as drops. Also, due to mechanical loading and fluid flow within the joint, the accessibility of nutrients to the cells is expected to be better. However, before this therapy could be used to cure patients, a number of factors have to be addressed. The amount and proliferative capacity of human and bovine meniscus cells probably are different and age dependent [4, 24]. This should be investigated in separate experiments. Additionally, it is known that the best results of repairing meniscus tears are obtained when the intervention is performed shortly after the injury, when the tear is still fresh. However, in case of a degenerative tear the use of a surgical rasp would help to refresh the wound environment and exposing collagen fibers to enhance adhesion of the glues. Also the use of collagenase could help to reprogram the wound environment, thereby facilitating healing [12, 13]. The proposed treatment is primarily aimed at traumatic tears since those have the best healing capacity. Degenerative tears are, in general, much more difficult to treat due to both interrupted homeostasis of the knee, and the limited availability of cells to rebuild the matrix [25]. Therefore, the main focus of the proposed therapy is on the repair of traumatic meniscus tears.

## Conclusions

Modification of the surface of meniscus tissue by incubation in collagenase and the presence of growth factor TGF-β3 were shown to have a positive influence on the number of cells present in the gap of a meniscus repair construct glued with novel reactive isocyanate-terminated three-armed- and hyper-branched glues. The healing potential of a torn meniscus can possibly be enhanced by using a combined therapy which makes use of biodegradable glues, collagenase tear surface treatment and the presence of growth factors. Further longer-term in vitro and in vivo studies are needed to validate these findings.

## References

[1] Simson JA, Strehin IA, Allen BW, Elisseeff JH (2013). Bonding and fusion of meniscus fibrocartilage using a novel chondroitin sulfate bone marrow tissue adhesive. Tissue Eng Part A.

[2] Ishimura M, Ohgushi H, Habata T, Tamai S, Fujisawa Y (1997). Arthroscopic meniscal repair using fibrin glue .2. Clinical applications. Arthroscopy.

[3] Scotti C, Pozzi A, Mangiavini L, Vitari F, Boschetti F, Domeneghini C (2009). Healing of meniscal tissue by cellular fibrin glue: an in vivo study. Knee Surg Sports Traumatol Arthrosc.

[4] Chevrier A, Nelea M, Hurtig MB, Hoemann CD, Buschmann MD (2009). Meniscus structure in human, sheep, and rabbit for animal models of meniscus repair. J Orthop Res.

[5] Arnoczky SP, Warren RF (1982). Microvasculature of the human meniscus. Am J Sports Med.

[6] Okuda K, Ochi M, Shu N, Uchio Y (1999). Meniscal rasping for repair of meniscal tear in the avascular zone. Arthroscopy.

[7] Uchio Y, Ochi M, Adachi N, Kawasaki K, Iwasa J (2003). Results of rasping of meniscal tears with and without anterior cruciate ligament injury as evaluated by second-look arthroscopy. Arthroscopy.

[8] Fox JM, Rintz KG, Ferkel RD (1993). Trephination of incomplete meniscal tears. Arthroscopy.

[9] Zhang Z, Arnold JA, Williams T, McCann B (1995). Repairs by trephination and suturing of longitudinal injuries in the avascular area of the meniscus in goats. Am J Sports Med.

[10] Rubman MH, Noyes FR, Barber-Westin SD (1998). Arthroscopic repair of meniscal tears that extend into the avascular zone. A review of 198 single and complex tears. Am J Sports Med.

[11] Longo UG, Campi S, Romeo G, Spiezia F, Maffulli N, Denaro V (2012). Biological strategies to enhance healing of the avascular area of the meniscus. Stem Cells Int.

[12] Qu F, Lin J-MG, Esterhai JL, Fisher MB, Mauck RL (2013). Biomaterial-mediated delivery of degradative enzymes to improve meniscus integration and repair. Acta Biomater.

[13] Qu F, Pintauro MP, Haughan JE, Henning EA, Esterhai JL, Schaer TP (2015). Repair of dense connective tissues via biomaterial-mediated matrix reprogramming of the wound interface. Biomaterials.

[14] Petersen W, Pufe T, Stärke C, Fuchs T, Kopf S, Neumann W (2007). The effect of locally applied vascular endothelial growth factor on meniscus healing: Gross and histological findings. Arch Orthop Trauma Surg.

[15] Ionescu LC, Lee GC, Huang KL, Mauck RL (2012). Growth factor supplementation improves native and engineered meniscus repair in vitro. Acta Biomater.

[16] Cucchiarini M, Schmidt K, Frisch J, Kohn D, Madry H (2015). Overexpression of TGF-β via rAAV-Mediated gene transfer promotes the healing of human meniscal lesions ex vivo on explanted menisci. Am J Sports Med.

[17] Bochyńska AI, Van Tienen TG, Hannink G, Buma P, Grijpma DW (2016). Development of biodegradable hyper-branched tissue adhesives for the repair of meniscus tears. Acta Biomater.

[18] Bochyńska AI, Sharifi S, van Tienen TG, Buma P, Grijpma DW (2013). Development of Tissue Adhesives Based on Amphiphilic Isocyanate-Terminated Trimethylene Carbonate Block Copolymers. Macromol Symp.

[19] Hennerbichler A, Moutos FT, Hennerbichler D, Weinberg JB, Guilak F (2007). Interleukin-1 and tumor necrosis factor alpha inhibit repair of the porcine meniscus in vitro. Osteoarthr Cartilage.

[20] Hennerbichler A, Moutos FT, Hennerbichler D, Weinberg JB, Guilak F (2007). Repair response of the inner and outer regions of the porcine meniscus in vitro. Am J Sports Med.

[21] Sporn M, Roberts A, Wakefield L, Assoian R (1986). Transforming growth factor-beta: biological function and chemical structure. Science.

[22] van de Breevaart Bravenboer J, In der Maur CD, Bos PK, Feenstra L, Verhaar JA, Weinans H (2004). Improved cartilage integration and interfacial strength after enzymatic treatment in a cartilage transplantation model. Arthritis Res Ther.

[23] Janssen LM, In der Maur CD, Bos PK, Hardillo JA, van Osch GJ (2006). Short-duration enzymatic treatment promotes integration of a cartilage graft in a defect. Ann Otol Rhinol Laryngol.

[24] Ionescu LC, Lee GC, Garcia GH, Zachry TL, Shah RP, Sennett BJ (2011). Maturation state-dependent alterations in meniscus integration: implications for scaffold design and tissue engineering. Tissue Eng Part A.

[25] Bochyńska AI, Hannink G, Grijpma DW, Buma P (2016). Tissue adhesives for meniscus tear repair: an overview of current advances and prospects for future clinical solutions. J Mater Sci Mater Med.

